# Local auxin synthesis mediated by YUCCA4 induced during root-knot nematode infection positively regulates gall growth and nematode development

**DOI:** 10.3389/fpls.2022.1019427

**Published:** 2022-11-16

**Authors:** Reira Suzuki, Yuri Kanno, Patricia Abril-Urias, Mitsunori Seo, Carolina Escobar, Allen Yi-Lun Tsai, Shinichiro Sawa

**Affiliations:** ^1^ Faculty of Advanced Science and Technology, Kumamoto University, Kumamoto, Japan; ^2^ International Research Organization for Advanced Science and Technology (IROAST), Kumamoto University, Kumamoto, Japan; ^3^ Dormancy and Adaptation Research Unit, RIKEN Center for Sustainable Resource Science, Yokohama, Kanagawa, Japan; ^4^ Facultad de Ciencias Ambientales y Bioquímica, Área de Fisiología Vegetal, Universidad de Castilla-La Mancha, Toledo, Spain; ^5^ International Research Center for Agricultural & Environmental Biology, Kumamoto University, Kumamoto, Japan

**Keywords:** YUC genes, auxin synthesis, meloidogyne incoginta, heterodera schachtii, phytohormomes, plant parasitic nematodes

## Abstract

Parasites and pathogens are known to manipulate the host’s endogenous signaling pathways to facilitate the infection process. In particular, plant-parasitic root-knot nematodes (RKN) are known to elicit auxin response at the infection sites, to aid the development of root galls as feeding sites for the parasites. Here we describe the role of local auxin synthesis induced during RKN infection. Exogenous application of auxin synthesis inhibitors decreased RKN gall formation rates, gall size and auxin response in galls, while auxin and auxin analogues produced the opposite effects, re-enforcing the notion that auxin positively regulates RKN gall formation. Among the auxin biosynthesis enzymes, *YUCCA4* (*YUC4*) was found to be dramatically up-regulated during RKN infection, suggesting it may be a major contributor to the auxin accumulation during gall formation. However, *yuc4-1* showed only very transient decrease in gall auxin levels and did not show significant changes in RKN infection rates, implying the loss of *YUC4* is likely compensated by other auxin sources. Nevertheless, *yuc4-1* plants produced significantly smaller galls with fewer mature females and egg masses, confirming that auxin synthesized by *YUC4* is required for proper gall formation and RKN development within. Interestingly, *YUC4* promoter was also activated during cyst nematode infection. These lines of evidence imply auxin biosynthesis from multiple sources, one of them being YUC4, is induced upon plant endoparasitic nematode invasion and likely contribute to their infections. The coordination of these different auxins adds another layer of complexity of hormonal regulations during plant parasitic nematode interaction.

## Introduction

Root-knot nematodes (*Meloidogyne incognita*, RKN) are plant parasites that cause significant damages to crop plants in many regions of the world. RKN infect a wide range of plant species, though are particularly prominent in solanaceae crops such as tomato and eggplant. RKN generation time takes 3~8 weeks depending on the environment. Juveniles molt once within the eggs and are hatched as infective second-instar juveniles (J2), which then seek out the roots of appropriate host plants to infect. Once a suitable host has been found, J2 enter the roots and migrate toward the vasculature, where they induce the formation of specialized feeding organs known as root-knots or galls (Wyss et al., 1992; Caillaud et al., 2008). RKN J2 are known to inject various effector proteins into vascular cells, which induces their differentiation into giant cells (GC) to serve as the source of sustenance of the endophytic RKN. Typically a gall contains 5~7 GC, which make up the majority of the gall mass and volume (Bird, 1992; Jones and Payne, 1978). As the endophytic RKN feed on the GC, they continue to molt and mature. Eventually adult female RKN lay eggs on the root surface to continue the life cycle.

Similar to many other parasites, RKN utilize the host’s endogenous hormonal signaling pathways to modify the infection sites to their favor. One important phytohormone that regulates RKN gall formation is auxin, which is best known for its role in embryogenesis, organogenesis and tropism (Zhao, 2010). The link between auxin and RKN infections have in fact been documented extensively in the past (Favery et al., 2016; Gheysen and Mitchum, 2019). Exogenous auxin applications have been found to enhance *Meloidogyne javanica* infections in peach (*Prunus persica*) and tomato (*Solanum lycopersicum*) (Kochba and Samish, 1971; Glazer et al., 1986). Mutations or down-regulation of genes involved in auxin synthesis, transport and perception are associated with reduced RKN susceptibility in various plant species, suggesting auxin is essential during RKN infection (Richardson and Price, 1984; Kyndt et al., 2016; Wang et al., 2018). Specifically, auxin has been shown to positively regulate GC growth, cell wall deformation around the GC and cell cycle activation within galls (de Almeida Engler et al., 1999). On the other hand, several auxin response factors and transporters were found to be strongly up-regulated during RKN infection, confirming that RKN actively stimulate auxin signaling at the site of infection (Cabrera et al., 2014; Kyndt et al., 2016; Olmo et al., 2020).

Auxins include a group of small molecules with an aromatic ring and a carboxylic acid functional group less than 0.55 Å apart (Sauer et al., 2013). Several naturally-occurring auxins have been detected in plants, though by far the most biologically significant and best characterized is indole-3-acetic acid (IAA). In addition, several synthetic auxins have been made as herbicides or plant growth stimulants, such as 1-naphthaleneacetic acid (NAA) and 2,4-dichlorophenoxyacetic acid (2,4-D). IAA is synthesized from the amino acid tryptophan (Trp) *via* indole-3-pyruvic acid (IPA). Trp is converted to IPA through the removal of the amino group catalyzed by TRYPTOPHAN AMINOTRANSFERASE OF ARABIDOPSIS (TAA) (Stepanova et al., 2008). IPA is then converted into IAA through decarboxylation catalyzed by the YUCCA (YUC) family of flavin-containing monooxygenases as the rate-limiting step of the auxin synthesis pathway (Mashiguchi et al., 2011; Won et al., 2011). Five TAA and 11 YUC homologues are present in the Arabidopsis genome (Matthes et al., 2018). The *TAA* genes are fairly ubiquitously expressed, whereas the *YUC* genes show more diverse expression patterns to synthesize auxins in different organs (Cheng et al., 2006). The *YUC* genes are thus the determining factors that dictate local auxin synthesis. Typically auxins are synthesized in the shoot then transported downward to the roots, and this auxin gradient plays important roles in apical-basal polarity in plants (Benková et al., 2003). However, auxin synthesis indeed also occur in roots, auxin made in the shoots were shown to not be sufficient to mediate root development (Chen et al., 2014).

Here we show that exogenous application of synthetic auxins and auxin synthesis inhibitors affect RKN infection frequency and resultant gall size. *YUC4*, which is typically associated with floral development, was found to be induced during RKN development. Although the *yuc4-1* mutation did not profoundly affect RKN infection rates, the numbers of emerging adult females and egg masses, gall size and gall IAA level were reduced in the *yuc4-1* mutant. Interestingly, the expression of *YUC4* was also induced after *Heterodera schachtii* infection, but loss of function *yuc4-1* plants showed no significant alteration of cyst nematode establishment or in the number of females or males *YUC4* appears to be specifically induced in roots upon RKN infection to facilitate gall development, even though *YUC4* is likely not the only source of auxin during RKN infection.

## Materials and methods

### Plant materials and growth conditions

Arabidopsis (*Arabidopsis thaliana*) of the ecotype Columbia-0 (Col-0) were used for this study. *yuc4-1* (SM_3_16128), *yuc1* (SALK_106293) and *pYUC::GUS* transgenic plants were described in Cheng et al., 2006 and Xu et al., 2017. Seeds were treated with 70% (v/v) ethanol, followed by surface sterilization [6% (w/v) antiformin, 0.04% (v/v) Triton X-100] for 10 minutes and 3 washes with ddH_2_O. Seeds were vernalized for 2 days and germinated on Murashige and Skoog (MS) media [1/4x MS salt (Sigma), 0.5% (w/v) sucrose, 0.6% (w/v) gellan gum, pH 6.4] in 23°C under constant light (70 µmol/sm^2^).

Auxin analogues and auxin synthesis inhibitors were dissolved in dimethyl sulfoxide (DMSO), then added to media at the following final concentrations: 2,4-D, IAA and NAA: 0.1 µM, L-kynurenine:10 µM, yucasin: 50 µM. Seedlings were transferred to media with hormones or auxin synthesis inhibitors 1 day after RKN inoculation for 24 hours, before returning to untreated MS media.

### Nematode infection assay

The maintenance, harvesting and sterilization of root-knot nematodes (*Meloidogyne incognita*) collected from Koshi city, Kumamoto prefecture, Japan were essentially performed as described in Nishiyama et al., 2015. Five day-old Arabidopsis seedlings were inoculated with RKN at 80 J2s per seedling, then incubated in 25°C under the short-day light cycle [8 hours light (70 µmol/sm^2^), 16 hours dark]. Galls were counted and diameters measured at 14 days post-inoculation (DPI), and egg masses were counted at 42 DPI. Diameter is defined as the longest distance across a gall perpendicular to the axis of the root.

Cyst nematodes (*Heterodera schachtii*; Austria) were kindly provided by J. Hofmann (BOKU University; Austria) and propagated in mustard roots (Sinapsis alba cv Albatros) grown in Gamborg medium (Gamborg et al., 1968) with 3% sucrose and 0.8% Daishin agar (pH 6.4) at 23°C in darkness as described by Bohlmann and Wieczorek (2015). Egg hatching was stimulated in 3 mM ZnCl_2_ (Bohlmann and Wieczorek, 2015). CN infections of Arabidopsis were performed as described in Olmo et al. (2017). 7 day-old seedlings were inoculated with 20-30 J2 per plant, root segments containing syncytia were hand-dissected for GUS assays at the infection stages indicated in the figures legend. The numbers of females and males in the *yuc4-1* plants were counted at 14 DPI under a stereomicroscope.

### Histological staining and microscopy

For RKN acid fuchsin staining, seedlings were harvested and washed with ddH_2_O, then cleared with 1% (v/v) antiformin for 5 minutes. Seedlings were then washed with ddH_2_O twice, and stained with 30 fold-diluted acid fuchsin [0.35% (w/v) acid fuchsin in 75% (v/v) acetic acid] at 100°C for 10 minutes. Cooled samples were washed twice with ddH_2_O, then de-stained with acidified glycerol (1.2 mM HCl in glycerol).

Promoter-GUS staining were performed essentially as described in Cabrera et al., 2014. Tissue samples were fixed in 90% (v/v) acetone for at least a day. Samples were washed with ddH_2_O then incubated in GUS staining solution {100 mM NaPO_4_, 10 mM ethylenediaminetetraacetic acid (EDTA) pH 8.0, K_3_[Fe(CN)_6_] and mM K₄[Fe(CN)] (0.5 mM for CN-infected plants, 3 mM for all other samples), 0.01% (w/v) Triton X-100, 0.5 mg/ml 5-bromo-4-chloro-3-indolyl-β-glucuronidase} in 37°C for 4 hours (uninfected and 1~7 DPI samples) or overnight (14~42 DPI samples). Reactions were stopped by incubating the samples 90% (v/v) ethanol and 10% (v/v) acetic acid. Samples were cleared and mounted using 80% chlorohydrate (w/v) in 33% (v/v) glycerol.

For toluidine blue-stained sections, tissues were vacuum-infiltrated with fixative [2% (v/v) glutaraldehyde, 0.02 M cacodylic acid] for 30 minutes, then incubated in 4°C overnight. Samples were washed for 10 minutes 5 times with 20 mM cacodylic acid, then dehydrated through an ethanol gradient (50%, 75%, 90%, 95% each 10 minutes, then 100% for 20 minutes). Samples were then transferred to a 1:1 ethanol:Technovit 7100 (Kulzer) mixture and shaken in room temperature for 2 hours, then transferred to a pre-embedding solution [1% (w/v) sulfurizing reagent I in Technovit 7100] and shaken overnight in room temperature. Samples were submerged in embedding solution [6.6% (v/v) sulfurizing reagent II in pre-embedding solution] then incubated in 40°C overnight. Sample blocks were sectioned to 3~5 µm thickness using a Leica RM2255 microtome, then mounted using ddH_2_O and incubated in 60°C overnight. Samples were stained in 0.025% (w/v) toluidine blue (Waldeck) and 0.04% neutral red for several minutes, then mounted with EUKITT (O. Kindler).

CN-infected syncytia were imaged with Nikon SMZ1000, Olympus SZX16 stereomicroscope, or Nikon eclipse 90i microscope. All other microscopy works were performed using an Axio Imager M1 stereomicroscope (Zeiss) mounted with a DP71 digital camera (Olympus).

### Hormone quantification

Extraction, purification and quantification of plant hormones were performed as described in Kanno et al. (2016).

### Transcript analysis

RNA were extracted from plant tissue using the RNeasy Plant Mini Kit (Qiagen), then cDNA were synthesized using the PrimeScript RT Master Mix (Takara) according to the manufacturers’ instructions. 100 ng of cDNA per sample were used for transcript quantification using the FastStart Essential DNA Green Master and the Light Cycler 480 system (Roche) using the PCR program of 95°C 5 minutes, then 95°C 10 seconds, 60°C 10 seconds, 72°C for 10 seconds. GLYCEROLALDEHYDE 3-PHOSPHATE DEHYDROGENASE (GADPH) was used as internal control. Primers used for PCR include ARF5 (forward: ATCTCAACGGATCCAAATCG, reverse: GGGTCTCAGCTCTCAGTTGG), YUC4 (forward: ACGCATCTGGTCTATGGAATG, reverse: CGGACTTGTACGCACTGG), TAA1 (forward: CCCCACTACACTCCCATCACTC, reverse: TCACCAATGCCCACCCAATAC), GADPH (forward: TTAGTCGCAACCTGAAGCCATC, reverse: TTCCACTGCTACTTGACCTTCG). The expression levels of frequently used normalizer genes such as actin or ribosomal genes are altered in developing galls and GCs in Arabidopsis (Barcala et al, 2010), whereas GAPDH primers have been used for qPCR analyses to produce data consistent with reporter-GUS and microarray results (Barcala et al., 2010; Yamaguchi et al., 2017; Díaz-Manzano et al., 2019). Therefore, GAPDH used as a normalizer produce robust transcript data for developing galls in Arabidopsis.

## Results

### Local auxin synthesis regulates gall development

**Figure 1:**
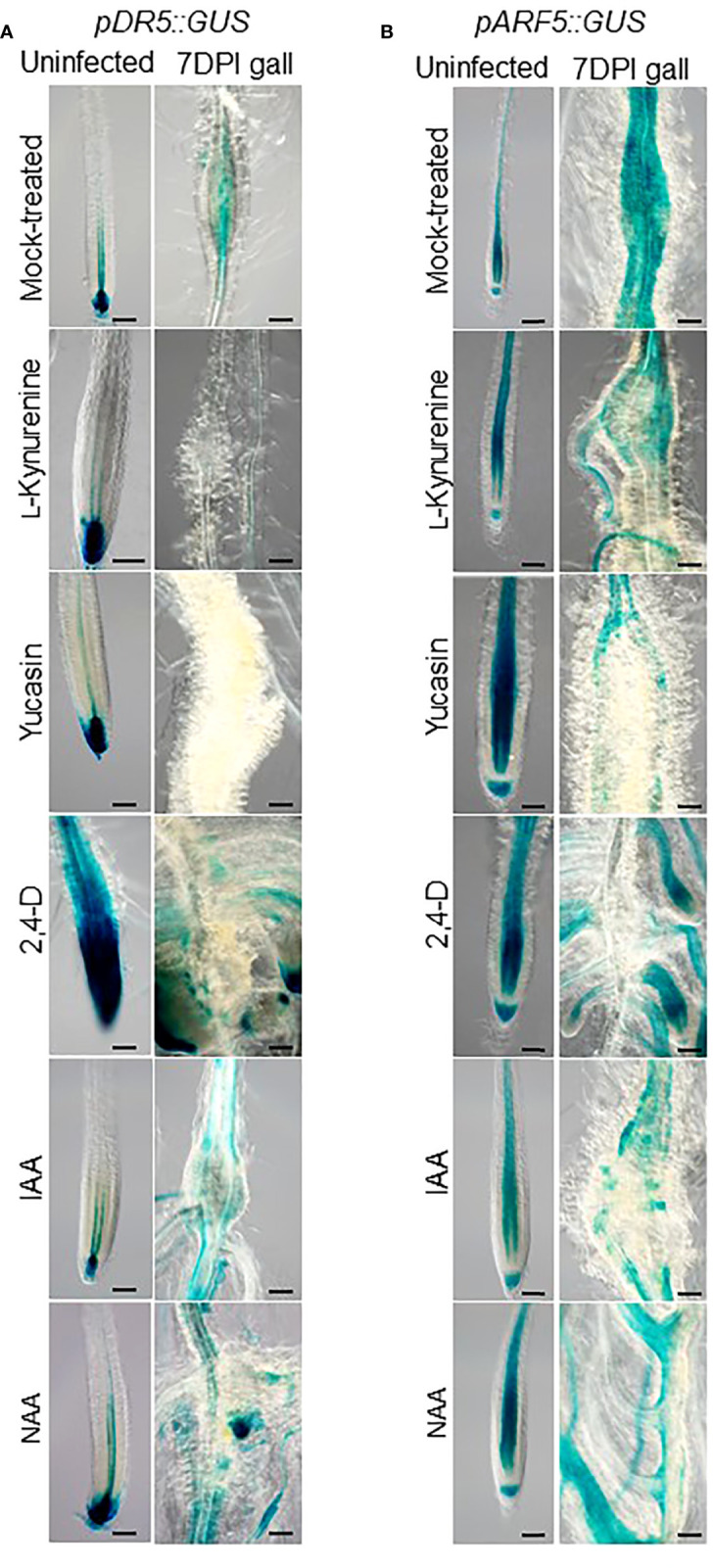
Localized auxin responses in galls alter in response to exogenous auxin treatments. Transgenic Arabidopsis seedlings with *pDR5::GUS* **(A)** or *pARF5::GUS* **(B)** reporter constructs that were mock (DMSO)-treated, treated with auxin synthesis inhibitor (10 µM L-kynurenine, 50 µM yucasin), or synthetic auxin analogues (0.1 µM 2,4-D, IAA or NAA), inoculated with RKN for 7 days or uninfected, then stained for GUS. Bar = 100 µm. At least three independent transgenic lines for each construct were examined with similar results.

In order to determine the role of auxin synthesis during RKN infection, Arabidopsis gall development was monitored in the presence of exogenous auxin or auxin synthesis inhibitors. L-kynurenine, the inhibitor of the enzyme TAA1 that converts Trp to IPA (He et al., 2011), and yucasin, the inhibitor of the enzyme YUC that converts IPA to IAA (Tsugafune et al., 2017), were used along with indole-3-acetic acid (IAA) and synthetic auxins 2, 4-dichlorophenoxy acetic acid (2,4-D) and naphthalene acetic acid (NAA) (Van Overbeek, 1959). To ensure these reagents exert their predicted effects, transgenic Arabidopsis plants with the *pDR5::GUS* and *pARF5::GUS* reporter constructs were used to monitor auxin accumulation and response patterns in RKN galls at 7 days post-inoculation (DPI). In the presence of auxin synthesis inhibitors, the *pDR5::GUS* signals were fully abolished in galls, and the *pARF5::GUS* signals were reduced to be found only in the vasculatures, even though the auxin maxima in the root tips remain unchanged (**Figure 1**). On the other hand, both *pDR5::GUS* and *pARF5::GUS* signals were found to be prominent in lateral roots when IAA and synthetic auxins were applied (**Figure 1**). These results confirm that dynamic auxin synthesis activities occur in galls during RKN infection.

**Figure 2:**
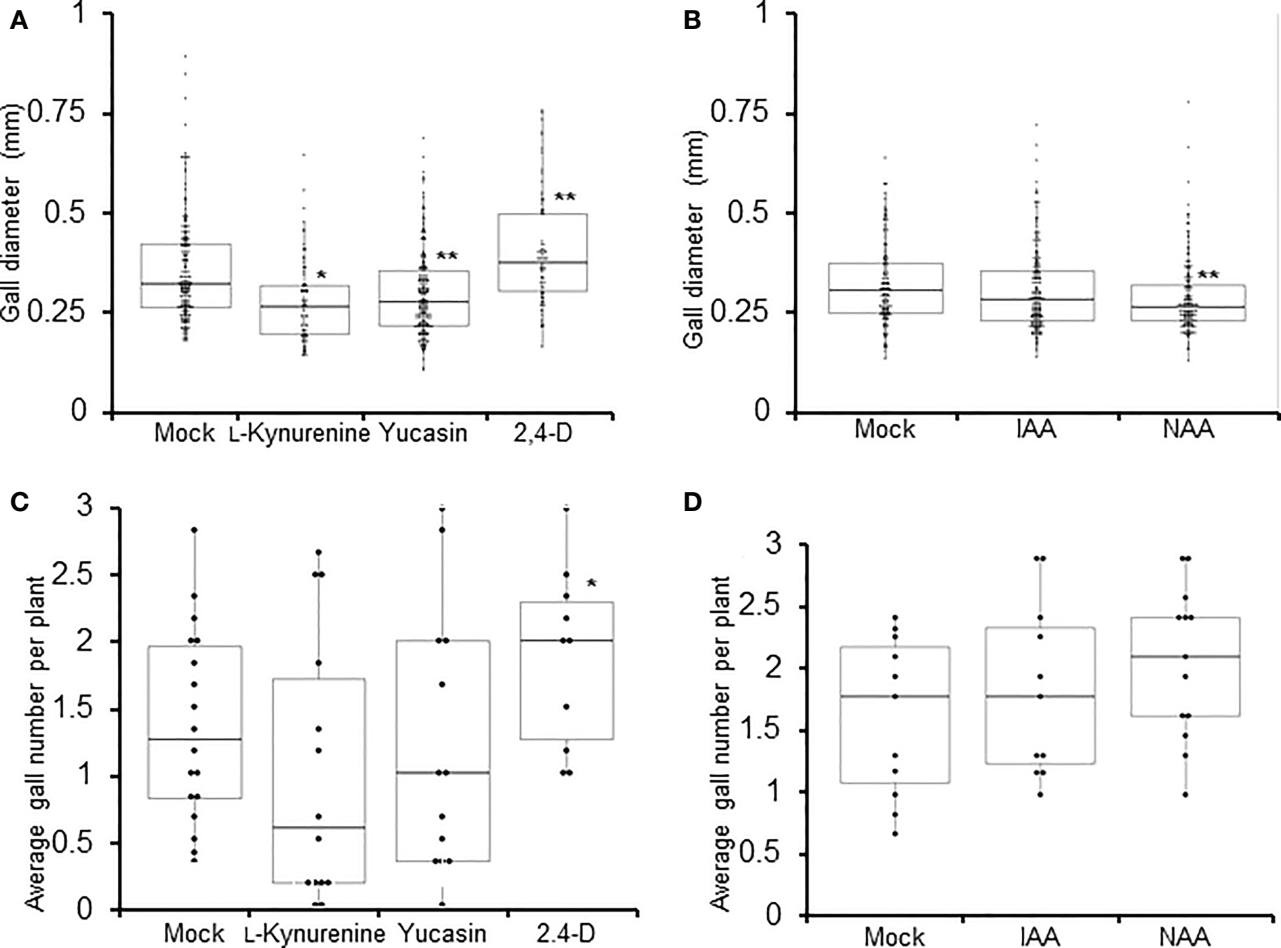
Auxin synthesis is required to maintain proper gall size. Gall diameters **(A, B)** and average gall numbers per plant **(C, D)** of Arabidopsis seedlings at 7 DPI treated with auxin synthesis inhibitors (L-kynyurenine, yucasin) or synthetic auxin analogues (2,4-D, IAA, NAA). Boxplots of n ≥ 77 ± SD **(A, B)**, and boxplots of n ≥ 14 **(C, D)** are shown. Each data point in **(C, D)** represents average value from 6 plants. * denotes significant difference from mock-treated samples, *P<0.05, **P<0.01, generalized Wilcoxon rank sum test corrected for multiple comparisons. At least three biological replicates were performed with similar results.

Knowing that auxin likely plays a role during RKN infection, the effect of auxin synthesis on the development of galls were evaluated next. L-kynurenine and yucasin treatments cause significant reductions in gall diameters, while 2,4-D treatment significantly increased gall diameters at 14 DPI (**Figure 2A**). Unexpectedly, IAA did not significantly affect gall size, while NAA actually significantly reduced gall diameter (**Figure 2B**). Different synthetic auxins with different structures are predicted to show variations in their physiological effects, and such has indeed been observed in above-ground organs (Abebie et al., 2008). It is conceivable that similar variations may occur in gall development as well. In addition, gall numbers in plants treated with auxin synthesis inhibitors and synthetic auxins were analyzed at 14 DPI. 2,4-D treatment was found to significantly increase gall numbers (**Figure 2C**), while L-kynurenine, yucasin, IAA and NAA did not significantly affect gall numbers (**Figure 2C, D**). Since both auxin synthesis inhibitors tested reduced gall size, local auxin synthesis may play a positive role during RKN infection. On the other hand, as neither of the auxin synthesis inhibitors tested affected gall numbers, locally synthesized auxin does not appear to affect the invasion of RKN into roots.

### Auxin synthetic gene YUC4 is expressed in galls

**Figure 3:**
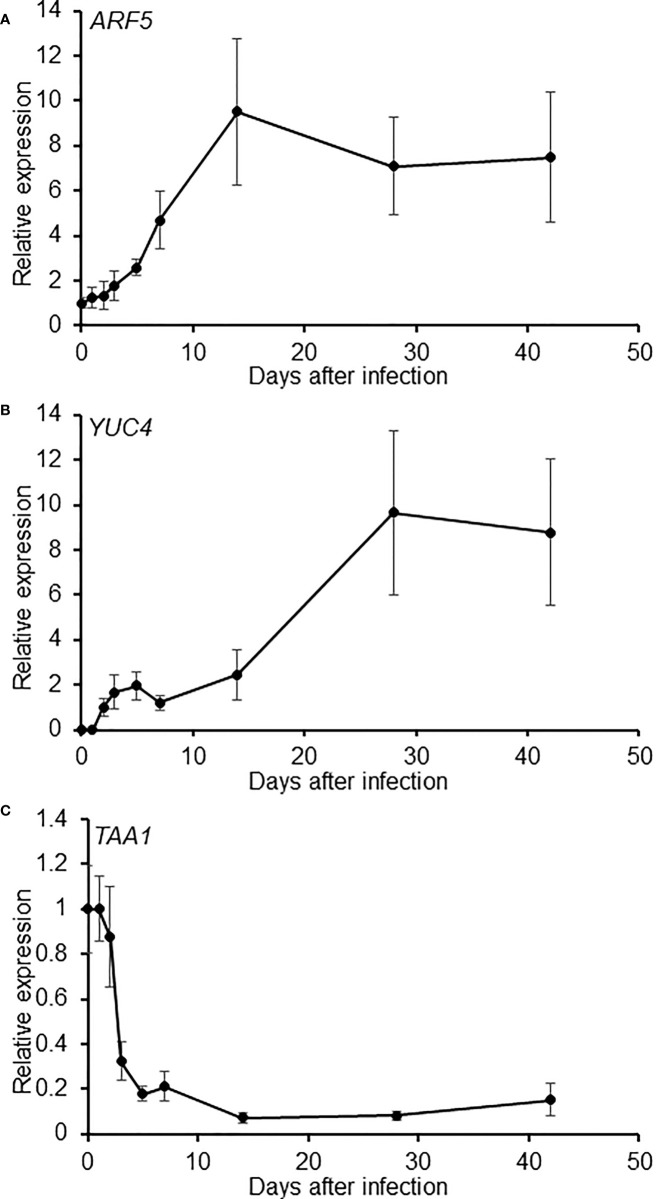
Expression of auxin metabolism genes are regulated during gall development. Relative expression levels of *ARF5* **(A)**, *YUC4* **(B)** and *TAA1* **(C)** quantified by qRT-PCR during gall development. Values were normalized to the earliest available time point. Averages of n = 9 ± SE are shown. Three biological replicates were performed with similar results.

**Figure 4:**
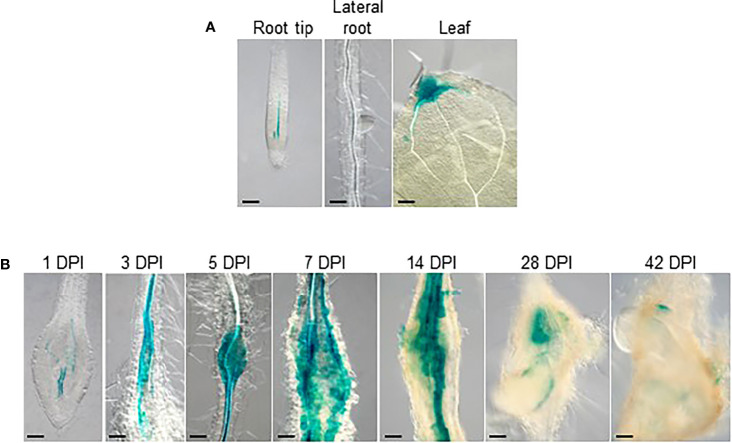
*YUC4* promoter activity is induced during gall formation. GUS-stained tissues of transgenic Arabidopsis with the *pYUC4::GUS* reporter construct. **(A)** Root tip, lateral roots and lea from uninfected plants, **(B)** 1-42 DPI galls. Bar = 100 µm. At least three independent transgenic lines were examined with similar results.

To further delineate the role of auxin during gall formation, the expression patterns of auxin synthesis genes were examined in developing galls. Using the transcriptome data from Yamaguchi et al., 2017, the expression patterns of auxin synthetic genes *TAA1* and the *YUC* genes were investigated over the first 7 days of RKN infection during RKN invasion and giant cell initiation. The expression levels of *TAA1* and most of the *YUC* genes remain relatively stable over the first 7 days of infection, except *YUC4* which was induced upon infection (**Supplemental Figure 1**). To more comprehensively determine the role of *YUC4* throughout RKN infection, the expression levels of *YUC4* and *TAA1* were examined by qRT-PCR over the entire course of RKN infection for up to 42 DPI. In addition, *AUXIN RESPONSE FACTOR 5* (*ARF5*), an auxin response signaling component known to be induced by and positively regulates *Meloidogyne javanica* infection was also analyzed (Olmo et al., 2020). *ARF5* transcript levels were found to rise fairly early at around 14 DPI and remained stable afterward, consistent with its expression pattern during *M. javanica* infection (**Figure 3A**, Olmo et al., 2020). On the other hand, *YUC4* transcript level increased modestly during early gall development, then rose prominently after 14 DPI (**Figure 3B**). On the contrary, *TAA1* transcript level decreased rapidly after RKN infection, and remained relatively low after 10 DPI (**Figure 3C**). To further confirm the expression pattern of *YUC4*, transgenic plant with the *pYUC4::GUS* reporter construct was established. In the absence of RKN infection, the *YUC4* promoter is active in the root tip, lateral roots and the distal ends of leaves (**Figure 4A**). Consistent with the transcriptome and qRT-PCR results, the *YUC4* promoter activity gradually increased during gall formation (**Figure 4B**). *pYUC4::GUS* signals became difficult to observe beyond 28 DPI, although this may be because the GUS signals were present only in the centers of galls and are not visible externally (**Figure 4B**). These lines of evidence confirm that *YUC4* is clearly induced by RKN infection, though *YUC4* transcripts appear to accumulate relatively slow compare to fast responders such as *ARF5*.

Considering YUC4 synthesizes auxin while ARF5 responds to auxin, and that *ARF5* is also significantly up-regulated during parasitic nematode infections (Olmo et al., 2020), the possibility that *YUC4* regulates *ARF5* expression during RKN infection was investigated. *ARF5* transcript levels were quantified before and after RKN infection in Col-0 and the *yuc4-1*loss-of-function mutant (Cheng et al., 2006; Xu et al., 2017). However, there were no significant differences in *ARF5* transcript levels between Col-0 and *yuc4-1* plants (**Supplemental Figure 2**). Together with the finding that *ARF5* gets up-regulated before *YUC4* upon RKN infection (**Figure 3**), this implies *YUC4* is unlikely to mediate the up-regulation of *ARF5* during RKN infection.

### *YUC4* positively regulates RKN maturation and fecundity

**Figure 5:**
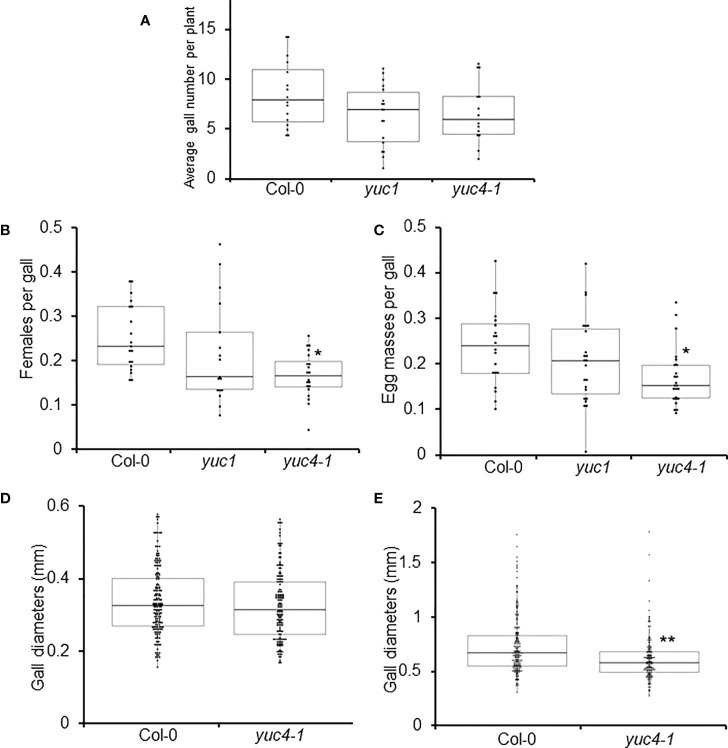
*YUC4* positively regulates RKN development and fecundity. Average gall numbers per plant at 14 DPI **(A)**, emerged RKN adult females per gall **(B)**, and egg masses per gall **(C)** in Col-0, *yuc1* and *yuc4-1* plants. **(D, E)** Diameters of 7 DPI **(D)** and 42 DPI galls **(E)** in Col-0 and *yuc4-1* plants. Each data point in **(A)** represents average value from 6 plants. Boxplots of n ≥ 204 are shown. Three biological replicates were performed with similar results. Asterisks denotes significance from Col-0, *P<0.05, **P<0.01, generalized Wilcoxon rank sum test corrected for multiple comparisons.

To further evaluate the function of *YUC4* during gall formation, RKN infection assays were performed using *yuc4-1*. For comparison, the *YUC4* homologue *YUCCA1* (*YUC1*), which is known to not be strongly up-regulated during RKN infection, was also analyzed (Cheng et al., 2006). No significant differences were detected in the numbers of galls, emerged mature females and egg masses from *yuc1* when compared to Col-0 (**Figures 5A–C**). Interestingly, the numbers of emerged mature females and egg masses reduced significantly in *yuc4-1* (**Figures 5B, C**), even though gall numbers remain unchanged (**Figure 5A**). This suggests *YUC4* likely regulates late-stage RKN development within galls, but not the initial gall formation during early infection, unlike *ARF5* which is known to prominently regulate gall formation frequency (Olmo et al., 2020). Furthermore, although no significant difference in gall diameters could be detected in *yuc4-1* at 7 DPI (**Figure 5D**), *yuc4-1* gall diameters were significantly reduced compared to that of Col-0 at 42 DPI (**Figure 5E**). The timing of *ARF5* and *YUC4* up-regulation induced by RKN infection appear to reflect their respective functions, where *ARF5* acts early to regulate gall initiation while *YUC4* acts late to promote gall growth and RKN maturation. The fact that *YUC4* does not transcriptionally regulate *ARF5* suggests these two genes likely function independent of each other during RKN infection.

### Localized auxin synthesis is required for RKN maturation

One other hormone known to function in concert with auxin to regulate cell division and differentiation is cytokinin (Schaller et al., 2014; Di Mambro et al., 2017). Despite primarily known as a plant hormone, cytokinin has been shown to be secreted by cyst nematodes to mediate infections (Siddique et al., 2015). Similarly, cytokinin is also known to be secreted by RKN, although its role in infection has yet to be validated (De Meutter et al., 2003). Other plant hormones such as salicyclic acid, jasmonic acid and ethylene are known to be involved in plant pathogen response signaling, suggesting they are likely also involved in the responses toward plant parasitic nematodes (Kammerhofer et al., 2015; Molinari, 2016).

**Figure 6:**
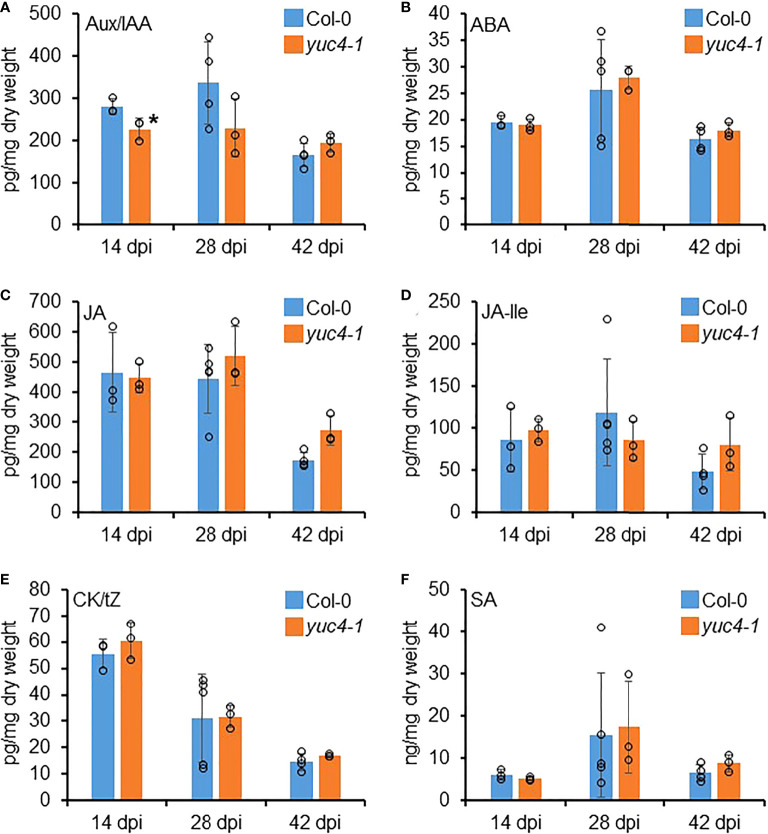
*YUC4* affects hormone levels in galls. Hormone levels of **(A)** auxin/indole-3-acetic acid (Aux/IAA), **(B)** abscisic acid (ABA), **(C)** jasmonic acid (JA), **(D)** JA isoleucine (JA-Ile), **(E)** cytokinine/trans-zeatin (CK/tZ) and **(F)** salicyclic acid (SA) in Col-0 and *yuc4-1* 14, 28 and 42 DPI galls. Averages from n ≥ 3 ± SD are shown, circles denote individual data points, * denotes significant difference from Col-0, P<0.05, Student’s T-test. Three biological replicates were performed with similar results. Data from 0~14 DPI are shown in **Supplemental Figure 3**.

To determine whether the *yuc4-1* RKN infection phenotypes are associated with reduced auxin synthesis, and whether other phytohormones are also involved with the *yuc4-1* gall development defects, phyotohormone levels were quantified in the Col-0 and *yuc4-1* galls at 14, 28 and 42 DPI where *YUC4* is highly expressed (**Figure 3B**). Auxin/indole-3-acetic acid (Aux/IAA), abscisic acid (ABA), jasmonic acid (JA), JA isoleucine (JA-lle), cytokinin/trans-zeatin (CK/tZ) and salicyclic acid (SA) levels were measured. *yuc4-1* galls showed a modest but significant reduction in Aux/IAA at 14 DPI as compared to the Col-0 (**Figure 6A**). However, no significant differences were detected in the levels of ABA, JA, JA-Ile and CK/tZ between Col-0 and *yuc4-1* galls at 14 to 42 DPI (**Figures 6B–F**). Also, no significant differences in these hormone levels were detected between Col-0 and *yuc4-1* from 1~14 DPI, where *YUC4* is not as strongly expressed (**Supplemental Figure 3**). Therefore *YUC4* does not appear to strongly affect phytohormone levels during gall formation, except auxin at around 14 DPI. Nevertheless, the fact that *yuc4-1* galls show reduced auxin level in galls at 14 DPI suggests the RKN maturation defects observed in *yuc4-1* may indeed be caused by reduced local auxin biosynthesis.

### *YUC4* is induced by cyst nematode *Heterodera schachtii* infection

**Figure 7:**
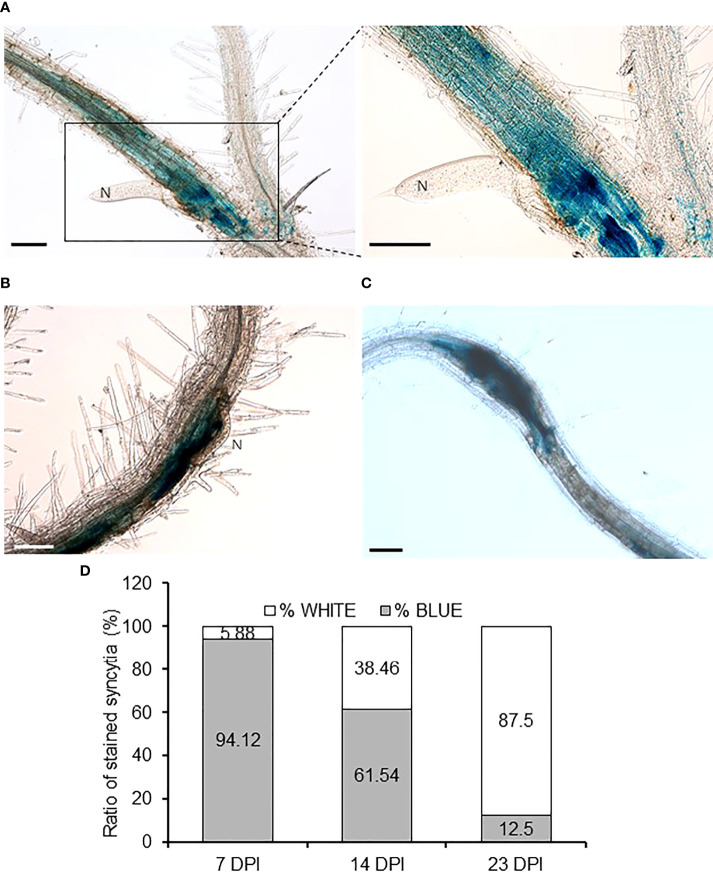
YUC4 promoter is active in Arabidopsis syncytia induced by *Heterodera schachtii*. **(A–C)** Roots of transgenic *pYUC4::GUS* Arabidopsis plants infected by *H. schachtii* at 7 DPI (right panel shows enlarged image of the syncytia) **(A)**, 14 DPI **(B)** and 23 DPI **(C)** showing intense GUS staining. Bars = 100 µm. **(D)** Percentages of GUS-positive syncytia from *pYUC4::GUS* plants at 7, 14 and 23 DPI. Three independent experiments were performed with at least 13 independent plants tested per infection point. Chi-square analysis [χ² (2, 46) = 22.53] indicated that the distribution of blue and white galls is significantly different among the three infection stages, P<0.05.

Considering the importance of *YUC4* in RKN infections, we also checked its putative role after the infection of another main group of plant endoparasitic nematodes, the cyst nematodes (CN, *Heterodera schachtii*). In CN-infected *pYUC4::GUS* transgenic Arabidopsis plants, *YUC4* promoter activity was clearly detected and localized within the syncytia with a high percentage of GUS-positive syncytia at 7DPI (**Figures 7A–D**). *YUC4* promoter activities remained with a detectable signal in syncytia at 14 and 23 DPI, although the percentage of GUS-positive syncytia decreased significantly over time (**Figures 7B–D**; p<0.05). This confirms that CN infection also induces *YUC4* expression in syncytia similar to the galls in the RKN infection, although *YUC4* promoter activity appears to decrease over the course of CN infection, in contrast to the slow increase during RKN infection.

To determine whether *YUC4* function was also crucial during CN infection similar to RKN, the numbers of males and females per plant were determined in *yuc4-1* plants as compared to that of the Col-0 control. Both, the number of males or females per plant or the total number of males plus females did not show significant differences between Col-0 and *yuc4-1* (**Supplemental Figure 4**). Thus, *YUC4* function does not strongly regulate CN establishment, sex determination or syncytia formation within the host plants. However, we cannot preclude a partial contribution of *YUC4* together with other YUCCA family members

## Discussion

Although auxin is a phytohormone best known for its roles in organogenesis and tropism, auxin plays a surprisingly critical role during plant-microbe interaction, pathogenesis and parasitism. For example, auxin has been implied to accumulate in the infection sites of protist *Plasmodiophora brassicae*, hemi-parasitic plant *Phtheirospermum japonicum* and bacteria *Xanthomonas oryzae* pv *oryzae* (Ding et al., 2008; Wakatake et al., 2020; Wang et al., 2022). Furthermore, *GmYUC2* was also found to regulate rhizobacteria root nodule formation in soybean (Wang et al., 2019). The importance of auxin can also be seen in plant-parasitic nematodes, as cyst nematodes similarly rely on auxin flow for proper syncytia formation (Grunewald et al., 2009). CN has been shown to induce auxin response in syncytia, possibly through the effector 10A07 to promote nuclear localization of the auxin master regulator IAA16 (Karczmarek et al., 2004; Hewezi et al., 2015). Our findings indicate that both RKNs and CNs infection induce *YUC4* in Arabidopsis, suggesting that it may be a common host plant response to the plant-parasitic nematodes (**Figure 3**, **Figure 7**). However, during CNs infection, *YUC4* may work with functionally-redundant genes (possibly other *YUC* genes) as *H. schachtii* infection, sex determination or syncytia formation was not altered in *yuc4-1* compared to Col-0 (**Figure 7**). In contrast, galls development was partially impaired in *yuc4-1* plants (**Figure 5**). Despite having superficially similar life cycles, RKNs and CNs feeding cells biology seems vastly distinct at the cellular and molecular level (Sijmons et al, 1991; Williamson and Gleason, 2003). Whereas RKN induce giant cell formation from repeated mitosis with partial cytokinesis of selected cells and lay eggs in egg masses, CN induce syncytia formation by fusing multiple host cells together and protect eggs within trauma-resilient cysts. In this respect, during CNs infection PIN-FORMED1 (PIN1)-mediated auxin efflux is needed to deliver auxins to the initial syncytial cell, where an auxin maximum is established. However, following initiation, PIN3 and PIN4 re-distribute the accumulated auxin laterally, which contribute to the radial expansion of the nematode feeding sites (Grunewald et al., 2009). In contrast, auxin importers such as AUXIN RESISTANT1 (AUX1) and LIKE AUX1 3 (LAX3) are needed not only for giant cell development but also for expansion (Kyndt et al., 2016). These differences may also explain the different responses of *yuc4-1* and *pYUC4::GUS* plants after CNs and RKNs infection. Further analysis may reveal details on the biological role of *YUC4*-mediated local auxin synthesis during CNs infection.

Auxin-responsive reporter activities could be detected in both early RKN and CN infection sites (Ganguly and Dasgupta, 1987; Hutangura et al., 1999; Karczmarek et al., 2004; Oosterbeek et al., 2021). In this study, we have directly quantified hormone levels in developing galls (**Figure 6**). Surprisingly, auxin level was reduced only modestly and transiently in *yuc4-1* (**Figure 6A**). It is possible that auxin accumulation is limited only in the giant cells and/or neighboring cells, such that the local auxin accumulation could be detected using reporter genes, but were masked when entire galls were examined. *pDR5:GFP* signals have been documented to be detected in early developing galls, but only in the neighboring cells and not in the giant cells, consistent with the notion that the overall auxin increase may not be apparent (Absmanner et al., 2013). In contrast, *pDR5:GUS* activity was detected in giant cells at 4DPI (Cabrera et al., 2014). GUS products are known to be very stable, therefore, it is also possible that the accumulated auxin in galls may been metabolized rapidly thus escaped detection.

Even though the effect of auxin in RKN infection has been characterized in the past, the specific effects of auxin synthesis inhibitors and auxin analogues appeared somewhat variable in our assays (**Figure 1**, **Figure 2**). Auxin synthesis inhibitors L-kynurenine and yucasin target different enzymes, therefore their effects cannot be expected to be identical. Simultaneous treatment with L-kynurenine and yucasin indeed have additive effects on seedling growth inhibition, confirming they utilize independent mechanisms to inhibit auxin synthesis (Nishimura et al., 2014). However, as auxin is an essential hormone with multiple synthesis pathways, it is physiologically impossible to fully abolish auxin synthesis. Even in the presence of synthesis inhibitors, background residual auxins always need to be accounted for. Similarly, auxin analogues IAA, NAA and 2,4-D also show varied effects on auxin distribution and RKN infection (**Figures 1**, **2**). Similar variations have been observed in rice floret closure and abscission reduction (Abebie et al., 2008; Huang et al., 2018). IAA, NAA and 2,4-D are known to bind the TIR1 receptor with distinct mechanisms due to their different sidechains (Tan et al., 2007). This is further complicated by the fact that there are multiple members of the TIR1/AFB family, each presumably have slightly different binding mechanisms to add further variations among auxin analogues (Parry et al., 2009). Lastly, there may be TIR1-independent auxin signaling pathway through auxin-binding proteins (ABP), which introduces variations among the responses of auxin analogues (Tromas et al., 2010). These lines of evidence suggest it may be difficult to define a concrete definitive auxin response based on the ligand alone.

The 11 *YUC* genes in the Arabidopsis genome have diverse expression patterns and structures to synthesize auxin in different organs (Kriechbaumer et al., 2017). The expression of *YUC* genes are also repressed by auxin in a negative feedback loop, further complicating their transcriptomic regulation (Suzuki et al., 2015). In general, auxin is primarily synthesized in the shoot, then moved toward the root through auxin transporters (Ljung et al., 2001). On the other hand, auxin synthesized in the shoot has been shown to be insufficient to rescue the auxin deficiency in roots, suggesting local auxin synthesis is also important in maintaining root growth (Yamada et al., 2009; Chen et al., 2014). In roots, gravitropism and metaxylem differentiation have been shown to be mediated by *YUC3*, *5*, *7*, *8* and *9* (Chen et al., 2014; Ursache et al., 2014). These *YUC* genes may account for the remainder of the local auxin synthesis in roots aside from *YUC4*, which may explain why very little differences were observed in the IAA levels of *yuc4-1* galls (**Figure 6A**). In contrast, *YUC4* is best known to regulate floral development along with *YUC1*, and their relationship with roots may not be immediately obvious (Cheng et al., 2006). *YUC4* and *YUC1* have been reported to regulate wounding-induced *de novo* organogenesis (Chen et al., 2016), which may partially explain why *YUC4* is up-regulated during RKN infection. *YUC4* is also unusual that it has 2 protein isoforms: YUC4.1 and YUC4.2. Only YUC4.2 possesses a transmembrane domain to anchor it to the ER cytosolic surface, and YUC4.2 is exclusively involved in flower development (Kriechbaumer et al., 2012). It would be interesting to determine which of the YUC4 isoforms (if not both) is involved in RKN gall development.

Here we showed that YUC4 involved in local auxin synthesis, gall formation and RKN maturation (**Figure 5**, **Figure 6A**). Several other gene products, particularly those involved in cell cycle regulation, has been shown to regulate gall development and RKN endophytic growth. Over-expressing several members of the cell cycle regulator Kip-Related Proteins (KRP) family members lead to smaller giant cells with fewer nuclei, consequently smaller galls, and reduced numbers of galls and egg masses (Vieira et al, 2013; Vieira and de Almeida Engler, 2015; Coelho et al., 2017). The development of RKN in these mutants were also visibly delayed, possibly due to the lack of nourishment from underdeveloped giant cells. Similarly, silencing the Arabidopsis cell cycle regulator cyclin-dependent kinase CDKA;1 also leads to reduced numbers of galls, egg masses, and delayed RKN development (Van de Cappelle et al., 2008). The RKN development defects in *yuc4-1* appear subtle compared to these cell cycle regulator mutants. Without completely abolishing local auxin synthesis and auxin flow into the roots, residual auxin may be sufficient to drive cell proliferation to maintain gall development, which in turn supports RKN development.

Much regarding the roles of phytohormones during pathogen infections remain to be explored. Aside from their expected roles in modulating plant development during pathogenesis, other unorthodox mechanisms may also be at work. Both auxin and cytokinin have been shown to possibly influence RKN directly. Cytokinin has been shown to stimulate RKN J2 stylet thrusting, thus promote foraging behaviors (Kirwa et al., 2018). While NAA has been shown to alter RKN J2 surface lipophilicity, suggesting auxin-binding proteins may be present on RKN cuticles (Akhkha et al., 2002). Both auxin and cytokinin have been documented to be secreted by the parasitic nematodes themselves, possibly as an aide to the infection process (De Meutter et al., 2003). Further research on both phytohormones and parasite biology will likely provide a better framework on how these mechanisms overlap.

## Data availability statement

The original contributions presented in the study are included in the article/**Supplementary Material**. Further inquiries can be directed to the corresponding author.

## Author contributions

RS, CE, and SS conceived and designed the experiments. RS, YK, PA-U performed the experiments. MS and CE provided resources. RS and AT wrote the manuscript. AT and SS revised the manuscript. SS procured funding. All authors contributed to the article and approved the submitted version.

## Funding

This work was supported by KAKENHI (21K19273, 20KK0135, 20H00422, 18H05487, and JPJSBP120223206) from Japan Society for the Promotion of Science to SS, as well as by the Spanish Government (PID2019-105924RB-I00 MCIN/AEI/10.13039/501100011033 and RED2018-102407-T) and the Castilla-La Mancha Government (SBPLY/17/180501/000287 and SBPLY/21/180501/000033) to CE. PA-U was a recipient of an FPI grant from the Ministry of Science and Innovation.

## Acknowledgments

We thank Y. Zhao (University of California San Diego) for providing the *pYUC4::GUS* transgenic Arabidopsis, M. Watahiki (Hokkaido University) for providing the *yuc4-1* and *yuc1* mutant seeds.

## Conflict of interest

The authors declare that the research was conducted in the absence of any commercial or financial relationships that could be construed as a potential conflict of interest.

## Publisher’s note

All claims expressed in this article are solely those of the authors and do not necessarily represent those of their affiliated organizations, or those of the publisher, the editors and the reviewers. Any product that may be evaluated in this article, or claim that may be made by its manufacturer, is not guaranteed or endorsed by the publisher.
