# Supplementary figures and images for: Local auxin synthesis mediated by YUCCA4 induced during root-knot nematode infection positively regulates gall growth and nematode development
Source: Front Plant Sci. 2022 Nov 16;13:1019427. doi: 10.3389/fpls.2022.1019427 (PMC9709418; doi:10.3389/fpls.2022.1019427)

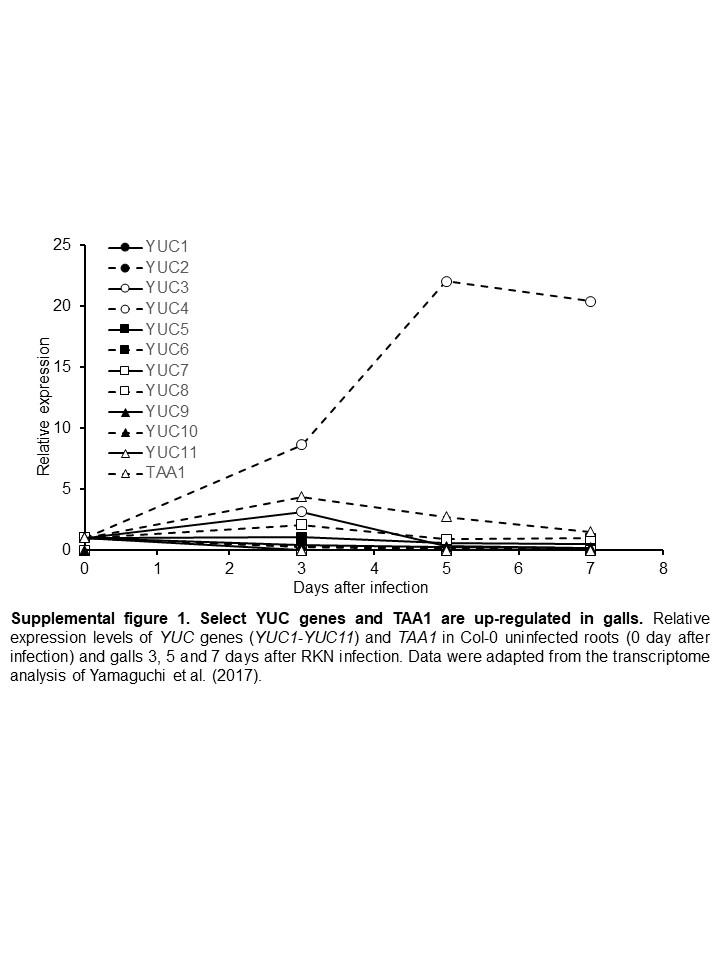

Supplement: Supplementary file 1 [file Image_1.jpeg]

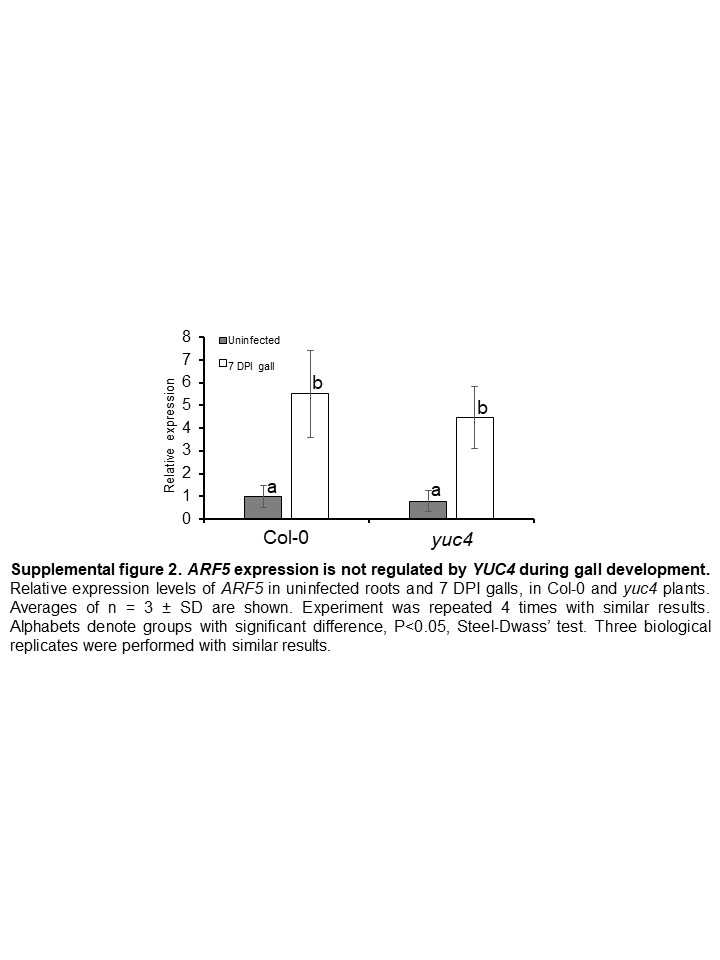

Supplement: Supplementary file 2 [file Image_2.jpeg]

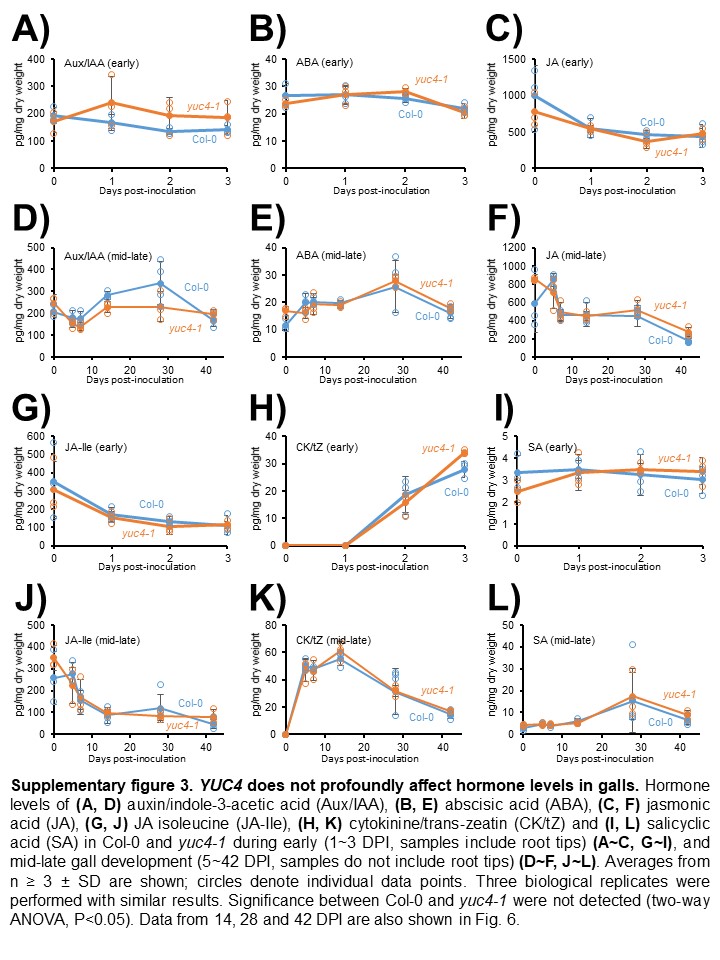

Supplement: Supplementary file 3 [file Image_3.jpeg]

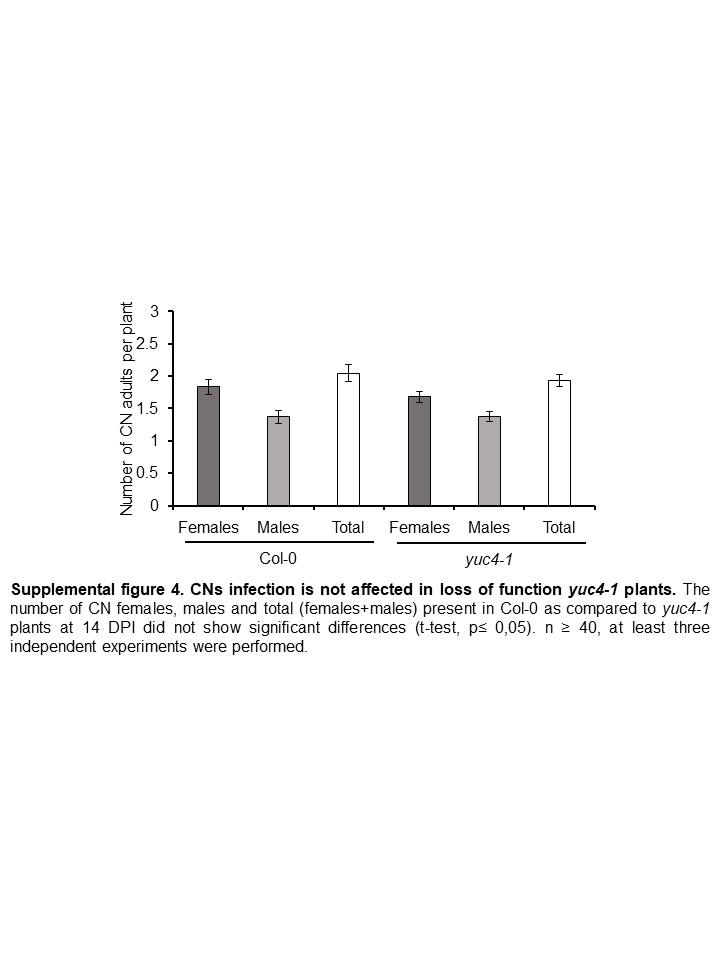

Supplement: Supplementary file 4 [file Image_4.jpeg]
